# Pure (acute) erythroid leukemia: morphology, immunophenotype, cytogenetics, mutations, treatment details, and survival data among 41 Mayo Clinic cases

**DOI:** 10.1038/s41408-022-00746-x

**Published:** 2022-11-02

**Authors:** Kaaren K. Reichard, Ayalew Tefferi, Maymona Abdelmagid, Attilio Orazi, Christina Alexandres, Joanna Haack, Patricia T. Greipp

**Affiliations:** 1 Department of Laboratory Medicine and Pathology – Division of Hematopathology; Mayo Clinic Rochester, Rochester, MN USA; 2 Division of Hematology; Mayo Clinic Rochester, Rochester, MN USA; 3 Department of Pathology, Texas Tech University Health Sciences Center, El Paso, TX USA; 4 Department of Neurology, Mayo Clinic Rochester, Rochester, MN USA

**Keywords:** Acute myeloid leukaemia, Acute myeloid leukaemia

## Abstract

Pure erythroid leukemia (PEL), also known as acute erythroid leukemia (AEL), is recognized as a distinct morphologic entity by both the 2016 and 2022 World Health Organization (WHO) classification system. By contrast, the 2022 International Consensus Classification (ICC) includes PEL under a broader category of “acute myeloid leukemia with mutated *TP53*”. We identified 41 Mayo Clinic cases of PEL (mean age 66 years, range 27–86; 71% males) and provide a comprehensive account of bone marrow morphology, immunophenotype, cytogenetic and mutation profiles. PEL was primary in 14 cases, therapy-related in 14, secondary in 12, and undetermined in one. All cases expressed biallelic *TP53* alterations, including *TP53* deletion/single *TP53* mutation (68%), two *TP53* mutations (29%) or two *TP53* deletions (3%); additional mutations were infrequent. Karyotype was complex in all cases and monosomal in 90%. Treatment details were available in 29 patients: hypomethylating agent (HMA) alone (*n* = 5), HMA + venetoclax (*n* = 12), intensive chemotherapy (*n* = 4), supportive care/other (*n* = 8); no responses or allogeneic stem cell transplants were documented, and all patients died at a median 1.8 months (range 0.2–9.3). The current study highlights a consistent and reproducible set of morphologic and genetic characteristics that identify PEL as a distinct AML variant whose dismal prognosis requires urgent attention.

## Introduction

Pure erythroid leukemia (PEL) is a rare and aggressive subtype of acute myeloid leukemia (AML) and accounts for approximately 1% of all AML diagnoses [1, 2]. While this diagnosis may seem relatively straightforward from the literature description, in clinical practice, in fact, PEL is often a diagnostic struggle even for the most seasoned of hematopathologists. In addition, pathologists may experience trepidation in rendering such a diagnosis given the gravity of a reported approximately <6 month overall median survival. Originally described by DiGuglielmo, numerous investigators, over the last several decades, have subsequently studied PEL and further refined its distinctive clinicopathologic features. PEL previously was a diagnostic subcategory in the revised fourth edition of the WHO classification [1, 2]. Currently PEL is classified as acute myeloid leukemia with mutated *TP53* in the International Consensus Classification [3] and as acute erythroid leukemia in the 5th edition of the WHO [4].

PEL is typically a disease of adults with median age at presentation of 66–68 years and its characteristics include complex/monosomal karyotype, TP53 protein overexpression, and biallelic *TP53* mutations. PEL might present *de novo* or arise as progression from antecedent myelodysplastic syndrome, myeloproliferative neoplasm, or as a therapy-related neoplasm [1, 2., 5–8] While there are reports of PEL/erythroblastic sarcoma in the pediatric population, there is evidence that unique genetic features exist in pediatric PELs, and likely represent a distinctly different disease process from that of adults [9]. Given that PEL is a rare and often difficult to diagnose disease with dismal prognosis, we looked into our institutional experience with the disease, with the objective to provide a comprehensive account of its pathology and clinical outcome; to the best of our knowledge, the current work constitutes the largest series of PEL in adults, to date.

## Methods

### Selection of cases

We retrospectively identified bone marrow cases seen and diagnosed at Mayo Clinic Rochester with a diagnosis of pure erythroid leukemia (PEL) based on the revised fourth edition World Health Organization criteria from the period of June 2017 to May 2022 [1, 2]. Case inclusion criteria required morphology slides available for review to confirm the diagnosis of PEL, confirmatory immunohistochemical and/or flow cytometric studies, cytogenetics and next-generation sequencing targeting genes commonly mutated in myeloid disease.

### Immunophenotyping studies

(i) *Immunohistochemistry*. Immunohistochemical analysis was performed on 4 μm, formalin-fixed, paraffin-embedded sections in all cases. A broad immunohistochemical panel was utilized in the evaluation of the cases. Primary antibodies included CD34 (QBEnd/10, Leica (Novocastra)), CD117 (YR145, Cell Marque), CD71 (MRQ-48, Cell Marque), E-Cadherin (4A2C7, Life Technologies), Myeloperoxidase (Dako), Hemoglobin (Cell Marque), Glycophorin A (GA-R2, Ventana), CD61 and TP53 (DO-7, Ventana).
(ii) *Flow cytometry*. After isotonic erythrocyte lysis, flow cytometric immunophenotyping was performed on anticoagulated bone marrow aspirate specimens using previously described methods [10]. Samples were examined with flow cytometric immunophenotyping using two eight-color tubes containing antibodies from BD Biosciences (Tube1: CD13 PE, CD15 V450, CD16 APC-H7, CD33 PE-Cy7, CD34 PerCP-Cy5.5, CD45 V500, CD117 APC, HLA-DR FITC and Tube2: CD2 FITC, CD7 PE, CD34 PerCP-Cy5.5, CD36 APC, CD38 V450, CD45 V500, CD56 PE-Cy7, CD64 APC-H7). A total of 100, 000 events were collected per case. The data were analyzed using Kaluza software (Beckman-Coulter, Brea, CA) and/or Diva software (BD Biosciences).

### Cytogenetics

Giemsa-banded (G-banded) chromosome analysis was performed on bone marrow samples according to conventional methods. When available, at least 20 metaphases were analyzed. Karyotypes of G-banded chromosomes were described according to the 2020 International System of Human Cytogenetic Nomenclature [11]. Abnormal clones were defined as two or more cells with the same structural abnormality, the same extra chromosomes, or the presence of three or more cells with loss of the same chromosome. Although technically clonal, cases with –Y, –X, + 15, and +Y were not considered a pathologic clonal finding but regarded as an age-related phenomenon. A complex karyotype is defined as ≥ 3 structural and/or numerical abnormalities. Monosomal karyotype is defined by the presence of one single autosomal monosomy (AM) in association with at least one additional AM or one structural chromosomal abnormality (excluding core-binding factor acute myeloid leukemia and acute promyelocytic leukemia).

### Fluorescence in situ hybridization

Interphase fluorescence in situ hybridization (FISH) for *TP53* locus-specific probe was performed on bone marrow aspirate interphase nuclei using standard FISH pretreatment, hybridization, and fluorescence microscopy protocols. A total of 200 interphase nuclei were evaluated in each case, with 100 nuclei evaluated independently by two qualified clinical cytogenetic technologists and interpreted by a board-certified (American Board of Medical Genetics and Genomics) clinical cytogeneticist. The expected typical abnormal FISH signal pattern for *TP53* deletion is 1R2G.

### Next-generation sequencing

Targeted NGS was performed to detect gene mutations commonly found in myeloid hematologic malignancies. DNA is extracted from the peripheral blood or bone marrow sample and following library preparation by hybrid capture, subjected to next-generation sequencing (NGS) with post-sequencing analysis of tumor-associated mutations. Performance characteristics of NGS panel: Single base substitutions: accuracy >99%; reproducibility 100% (intra- and interassay); sensitivity 5–10% variant allele fraction with a minimum depth coverage of 250X; Insertion/deletion events: accuracy >99%; reproducibility 100% (intra- and interassay); sensitivity 5–10% variant allele fraction with a minimum depth coverage of 250X. NGS testing performed at Mayo Clinic Rochester includes 42 commonly mutated genes in the panel, including *TP53* exons 4–9. (*ANKRD26* (NM_014915.2) 5’UTR, exons 1–4, intron c.172, *ASXL1* (NM_015338.5) exons 10–13, *BCOR* (NM_001123385.1) exons 4–15, *CALR* (NM_004343.3) exon 9, *CBL* (NM_005188.3) intron 7 last 100 bps before start of exon 8, exon 8, intron 8, and exon 9, *CEBPA* (NM_004364.4) exon 1, *CSF3R* (NM_000760.3) exons 14 and 17, *DDX41* (NM_016222.2) exons 1–17, *DNMT3A* (NM_022552.4) exons 8–23, *ELANE* (NM_001972.2) exons 1–5, *ETNK1* (NM_018638.4) exons 2–5, *ETV6* (NM_001987.4) exons 3–8, *EZH2* (NM_004456.4) exons 2–20, *FLT3* (NM_004119.2) exons 14–20, *GATA1* (NM_002049.3) exons 2 and 4, *GATA2* (NM_001145661.1) exons 3–7, intron 5, c.1017 + 1–1017 + 730, *IDH1* (NM_005896.3) exon 4, *IDH2* (NM_002168.3) exon 4, *JAK2* (NM_004972.3) exons 12–16, *KDM6A* (UTX) (NM_021140.3) exons 1–29, *KIT* (NM_000222.2) exons 8–11 and 17, *KRAS* (NM_033360.3) exons 2–3, *MPL* (NM_005373.2) exons 10–12, *NPM1* (NM_002520.6) exons 9–11, to −30 before exon 11, *NRAS* (NM_002524.4) exons 2 and 3, *PHF6* (NM_001015877.1) exons 2–10, *PTPN11* (NM_002834.3) exons 3–4 and 12–13, *RAD21* (NM_006265.2) exons 1, 2, 4–7, 9–11, 13, 14, exon 10 flank 15 bp, *RUNX1* (NM_001001890.2) exons 1–6, intron 4 c.725–13 T > A and intron 5 c.886 + 1–4del, *SETBP1* (NM_015559.2) partial exon 4; amino acids 400–950, *SH2B3* (LNK) (NM_005475.2) exon 2–8, *SF3B1* (NM_012433.2) exons 13–16, *SRP72* (NM_006947.3) exons 6, 10, *SMC3* (NM_005445.3) exons 7, 8, 13, 17, 19, 21, 29, *SRSF2* (NM_003016.4) exons 1 and 2, *STAG2* (NM_001042750.1) exons 4–34, 12, 17 and 22 flank 15 bp, *TERT* (NM_198253.2) exons 2–16, *TET2* (NM_001127208.2) exons 3–11, *TP53* (NM_000546.4) exons 4–9, *U2AF1* (NM_001025203.1) exons 2, 6, and 8, *WT1* (NM_024426.2) exons 1–10, and *ZRSR2* (NM_005089.3) exons 1–11).

## Results

### Clinical characteristics

**Fig. 1:**
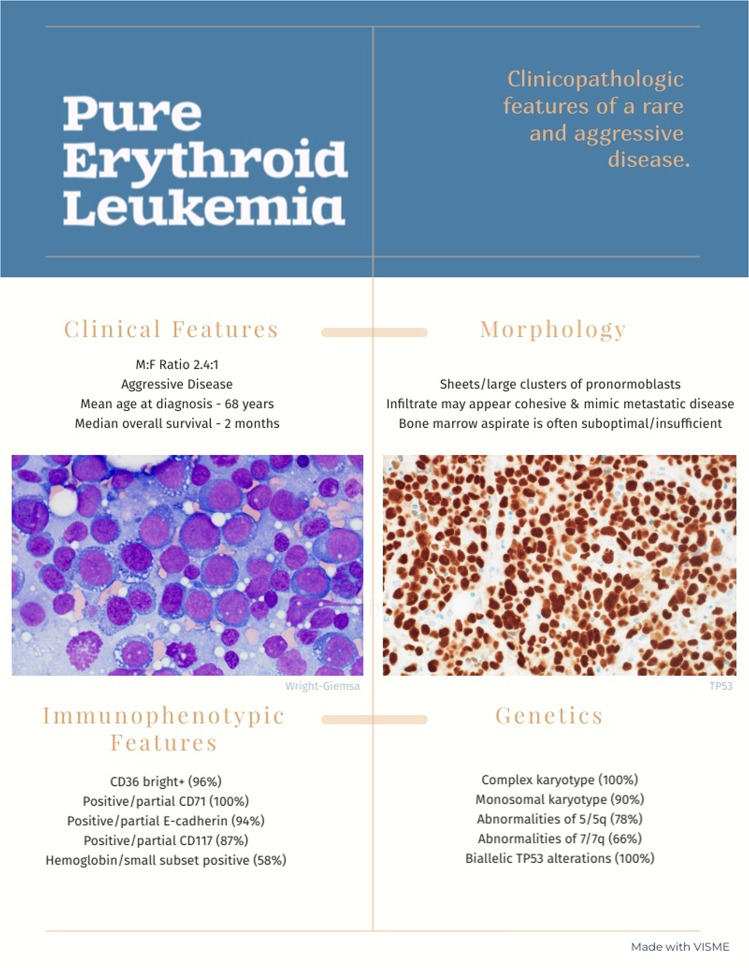
Pure erythroid leukemia - clinicopathologic features. Infographic depicting the key elements that are highly reproducible in pure erythroid leukemia.

We identified 41 cases of PEL that met our inclusion criteria (Fig. 1). There were 12 females and 29 males with a mean age of 66 years (range 27–86). As seen in Table 1, these cases included both secondary and *de novo* forms of PEL: 14 cases primary/de novo, 14 therapy-related, 12 secondary to progression of a myeloid disorder [MDS (5), MPN (5), MDS/MPN (2)], and one with no available clinical history. 100% of patients are dead of disease (41/41) with an overall mean survival of 3.3 months (median 2 months; range 0.1–23).

**Table 1:** Clinical, hematologic, bone marrow and immunophenotypic characteristics of 41 pure erythroid leukemia cases.

Variables	All patients
	*N* = 41
Age at diagnosis in years; mean (range)	68 (27–86)
Male, *n* (%)	29 (70.7%)
*Clinical presentation:*	
Primary, *n* (%)	14 (34%)
Therapy related, *n* (%)	14 (34%)
Secondary to MDS/MPN, *n* (%)	12 (29)
Unknown, *n* (%)	1 (2%)
Hemoglobin g/dL; mea*n* (range)	8.1 (5.9–11.3)
Anemia, *n* (%) *“N”* evaluable = 40	40 (100%)
Severe anemia, *n* (%) *“N”* evaluable = 40	19 (48%)
Leukocytes x 10^9^/L; mea*n* (range)	4.4 (0.6–23.9)
ANC x 10^9^/L; mea*n* (range)	1.9 (0–11.5)
Neutropenia, *n* (%) *“N”* evaluable = 40	25 (63%)
*Degree of neutropenia:*	
Marked, *n* (%)	10 (25%)
Moderate, *n* (%)	8 (20%)
Mild, *n* (%)	7 (18%)
Platelets × 10^9^/L; mea*n* (range)	32 (4–146)
Thrombocytopenia, *n* (%) *“N”* evaluable = 40	39 (98%)
*Degree of thrombocytopenia:*	
Marked	31 (78%)
Moderate	7 (18%)
Mild	1 (2%)
Circulating blasts, *n* (%) *“N”* evaluable = 40	27 (68%)
Circulating blast %; mea*n* (range)	11.5 (Rare-88)
>80% bone marrow erythroid precursors, *n* (%)	41 (100%)
>30% bone marrow pronormoblasts, *n* (%)	41 (100%)
Dysplastic megakaryocytes, *n* (%)	33 (80%)
Ring sideroblasts, *n* (%) *“N”* evaluable = 31	22 (71%)
Ring sideroblast %; mea*n* (range)	26 (Rare-80)
Carcinoma-like pattern, *n* (%)	8 (20%)
Intrasinusoidal pattern, *n* (%)	3 (7%)
*Immunophenotypic features by flow cytometry positive for; (%)*	
* CD45* (*“N”* evaluable =28)	Weak positive (50%).
* CD34* (*“N”* evaluable =28)	Positive (25%); partial positive (18%).
* CD117* (*“N”* evaluable =28)	Positive (61%); partial positive (21%); weak positive (4%).
* CD36* (*“N”* evaluable =28)	Positive (96%).
* HLA-DR* (*“N”* evaluable =28)	Positive (21%).
* CD13* (*“N”* evaluable =28)	Positive (43%); weak positive (4%); partial positive (21%).
* CD33* (*“N”* evaluable =28)	Positive (25%); partial positive (39%).
* CD7* (*“N”* evaluable =28)	Positive (36%); partial positive (39%).
* MPO* (*“N”* evaluable =15)	Positive (0%).
*Immunophenotypic features by Immunohistochemistry positive for; (%)*	
* CD34* (*“N”* evaluable =32)	Positive (16%); positive small subset (12%).
* CD117* (*“N”* evaluable =31)	Positive (68%); positive partial (19%); Weak (3%).
* CD71* (*“N”* evaluable =28)	Positive (96%); positive partial (4%).
* E-Cadherin* (*“N”* evaluable =33)	Positive (88%); positive partial (6%).
* Hemoglobin* (*“N”* evaluable =31)	Positive (0%); positive small subset (58%).
* Glycophorin A* (*“N”* evaluable =12)	Positive (0%); positive small subset (33%).
* CD61* (*“N”* evaluable =32)	Positive (0%); positive small subset (9%).
* AE1/AE3 keratin* (*“N”* evaluable =28)	Positive (0%)
* OSCAR* (*“N”* evaluable =17)	Positive (0%)
* MPO* (*“N”* evaluable =37)	Positive (0%)

### Hematologic indices and peripheral blood smear findings

All 40 patients with an available complete blood cell count presented with anemia (100%), essentially all with thrombocytopenia (98%) and 63% with neutropenia (Table 1). The average hemoglobin level was 8.1 g/dL with 48% of patients having severe anemia defined as <8.0 grams/deciliter. The average platelet count was 32 × 10(9)/L (range for-146) with 78% of patients demonstrating marked thrombocytopenia (defined as less than 50 × 10(9)/L). The average absolute neutrophil count was 1.9 × 10(9)/L (range 0–11.5). 25% of patients had marked neutropenia (<0.5 × 10(9)/L) 20% moderate neutropenia (0.5–1.0 × 10(9)/L); 18% mild neutropenia (1.01–1.5 × 10(9)/L). In those cases with sufficient numbers of neutrophils for cytologic evaluation, no cases showed significant dysplastic features (*N* = 21). There was an insufficient number of cases with sufficient platelets to accurately assess for platelet dysplasia. Circulating blasts were seen in 68% of cases with an average of 11.5% and a median of 4%.

### Bone marrow findings

Table 1 provides a list of the salient bone marrow findings. In 56% of cases (23/41), the bone marrow aspirate smears/touch preparations were of suboptimal/inadequate quality for cytologic review due to dry tap, hemodilution and/or numerous broken cells/bare nuclei. Therefore, an accurate differential count could not be performed in these cases and the assessment of bone marrow involvement by the neoplastic erythroid population was informed by the bone marrow core biopsy (Fig. 1). In all cases, greater than 80% of bone marrow nucleated elements consisted of erythroid precursors. For cases with an aspirate available, the percentage of proerythroblasts was ≥30% of all erythroblasts. 85% of all cases (35/41), including all cases with a dry tap aspirate, showed tumor cells predominantly at the pronormoblast stage of maturation. The remaining 15% of cases showed some minimal maturation past the pronormoblastic stage but no case showed a significant population of terminally differentiated nucleated red blood cell precursors. 80% of cases had dysplastic megakaryocytes characterized by small, monolobated forms and small forms with hyperchromatic nuclei. In all cases, there was insufficient granulopoiesis to assess for dysplasia and non-erythroid blasts were not increased. In 20% of the cases the pattern of infiltration by the pronormoblasts was cohesive/focal/nodular mimicking a metastatic tumor. 3 cases (7%) showed a distinctive sinusoidal pattern. Ring sideroblasts were detected in 71% of cases with an average percent of 26 (range; rare – 80).

### Flow cytometric findings

Twenty-eight of 41 cases (68%) had sufficient bone marrow aspirate material to identify the population of interest (pronormoblasts) (Fig. 1). Interestingly, we found that none of the tested antigens (except myeloperoxidase) showed a uniform presence or absence pattern of expressio*n* (see Table 1). The vast majority (96%) of pronormoblasts expressed bright CD36 and were negative for HLA-DR (79%). 75% of cases showed aberrant positivity for CD7 (36% uniform: 39% partial). CD45 was weakly expressed in exactly half of the cases. In terms of “blast” markers, CD117 was expressed in 82% of cases but only 61% showed uniform positive expression. CD34 was expressed in 25% of cases which is critical in considering the incorrect interpretation of a myeloid blast population since CD13 is expressed in 68% of cases (uniform positive 47%; partial positive 21%) and CD33 in 64% of cases (25% uniform positive; 39% partial positive). Myeloperoxidase, as would be expected, was uniformly negative.

### Immunohistochemical findings

Immunohistochemical studies were performed in a subset of cases, typically depending on the confidence of confirming erythroid lineage with an initial handful of markers (Table 1 and Fig. 1). The pronormoblasts in pure erythroid leukemia were essentially uniformly positive for CD71 (96%) with the majority being uniformly positive for CD117 (68%) and E-cadheri*n* (88%). A subset of cases only showed partial positivity for these markers (CD117 19%; CD71 4%; E-cadherin 6%). Although uncommon, the pronormoblasts may be negative for CD117 (10%) and E-cadheri*n* (6%). CD34 was uniformly positive in 16% of cases. Greater than 1/2 of the cases exhibited hemoglobin positivity in a subtle but convincing subset of blasts (58%) indicating that this marker is useful to confirm erythroid lineage when present. Similarly, glycophorin was convincingly positive in a small subset of pronormoblasts (33%). The majority of pronormoblasts /blasts were negative for CD61; however, 3 cases (9%) did show a distinctive small positive subset indicating that rare cases may show both erythroid and megakaryocytic differentiation.

### Cytogenetic characteristics

100% (41/41) demonstrated an abnormal karyotype (Table 2 and Fig. 1). Of the 41 cases, 73% met criteria for a highly abnormal karyotype (≥10 numerical and/or structural abnormalities) and 90% satisfied criteria for a monosomal karyotype. 78% of cases demonstrated a deletion abnormality involving chromosome 5/5q and 66% demonstrated a deletion abnormality involving chromosome 7/7q. Of the 41 cases, 29 (71%) demonstrated a presumed deletion of the *TP53* locus (17p13) of which 14 (34%) were confirmed by FISH.

**Table 2:** Genetic characteristics of 41 patients diagnosed with pure erythroid leukemia.

Variables	All patients
	*N* = 41
*Cytogenetics and FISH:*	
Abnormal karyotype, *n* (%)	41 (100%)
Highly abnormal karyotype, *n* (%)	30 (73%)
Monosomal karyotype, *n* (%)	37 (90%)
Deletion of chromosome 5/5q, *n* (%)	32 (78%)
Deletion of chromosome 7/7q, *n* (%)	27 (66%)
*TP53* (17p13) deletion by karyotype, (%)	29 (71%)
Confirmation of *TP53* deletion by FISH, (%)	14 (34%)
*Next-generation sequencing (42 myeloid gene panel):*	
Pathogenic *TP53* mutations:	
Single *TP53* mutation, *n* (%)	28 (68%)
Double *TP53* mutation, *n* (%)	12 (29%)
*Distribution of double TP53 mutations/deletions:*	
Double *TP53* deletions by karyotype, *n* (%)	1 (3%)
Double *TP53* mutations by NGS, *n* (%)	12 (29%)
*TP53* deletion and *TP53* mutation, *n* (%)	28 (68%)
Additional gene pathogenic mutations (VAF), *n* (%):	12 (29%)
* JAK2*V617F (secondary PEL from prior MPN), *n* (%)	5 (12%)
Patient 1	73%; *TP53* mutatio*n* (44%)
Patient 2	95%; *TP53* mutatio*n* (18%); also has *TET2* (41%)
Patient 3	57%; two *TP53* mutations (65% and 60%)
Patient 4	33%; *TP53* mutatio*n* (12%)
Patient 5	98%; two *TP53* mutations (92% and 85%); also has *IDH2* (47%) and *SRSF2* (46%)
* Other mutations:*	
* NRAS:* c.37 G > C; p.Gly13Arg	19%; two *TP53* mutations (83% and 75%)
* DNMT3A:* c.1848dup; p.Phe617Ilefs*2	9%; *TP53* 35%
* TET2:* c.4527_4531delinsC; p.Lys1509Asnfs*61	43%; *TP53* 53%
* TET2:* c.2400_2401del; p.His800Glnfs*15	41%; *TP53* 58%
* TET2:* c.4319_4329del; p.Arg1440Hisfs*34	37%; *TP53* 69%
* SF3B1:*c.2098 A > G; p.Lys700Glu	15%; two *TP53* mutations (42% and 38%)
* CSF3R:* c.2245 C > T; p.Gln749*	5%; two *TP53* mutations (28% and 38%)

### Mutations

40 of 41 (98%) cases demonstrated at least one *TP53* pathogenic mutation as detected by NGS (Table 2). In the single case with no *TP53* (or other) mutation detected by NGS, there were 2 *TP53* deletions detected by FISH. All 41 cases showed double *TP53* alterations as exemplified by either two *TP53* deletions by karyotype (3%; *N* = 1), double *TP53* mutations by NGS (29%; *N* = 12) or a combination of a *TP53* deletion and *TP53* mutatio*n* (68%; *N* = 28). Twelve patients (29%) showed additional pathogenic mutations in genes associated with myeloid disorders, in addition to the above-described *TP53* mutation(s). Therefore, an interesting overall finding is that aside from a single or double *TP53* mutation, the NGS findings are relatively “non-complex/simple” on a next-generation sequencing panel (42 genes in this study). Five of the 12 patients (42%) comprised disease progression from an underlying myeloproliferative neoplasm (MPN) and showed NGS evidence of a *JAK2* V617F mutation. The underlying MPN in these five patients included two cases of polycythemia vera, 2 cases of the essential thrombocythemia and 1 case favor primary myelofibrosis. Other pathogenic mutations that were detected in the non-MPN patients included abnormalities in several clonal hematopoiesis of indeterminate potential (CHIP) genes (e.g., *DNMT3A, TET2* and *SF3B1*); however, all exhibited variant allele frequencies greater than 20%, and some even higher in the 50% and 60% range. Of these non-*JAK2* mutation cases, the *NRAS*, *CSF3R* and *TET2* c.4319_4329del; p.Arg1440Hisfs*34 were in the context of therapy-related disease; the remaining 4 other mutations (*TET2, TET2, DNMT3A, and SF3B1*) were in the context of disease progression from myelodysplastic syndrome.

### Treatment details and survival

**Fig. 2:**
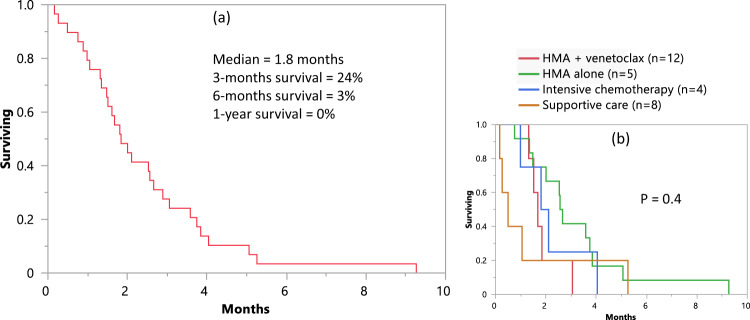
Pure erythroid leukemia - survival data. Overall survival data of 29 patients with pure erythroid leukemia (**a**), stratified by treatment received (**b**). HMA = hypomethylating agent; *One of 8 patients included in the supportive care category received quizartinib.

Treatment details were available in 29 patients seen and managed at the Mayo Clinic (Table 3). As was the case for the entire study population of 41 patients, all of these 29 patients expressed biallelic*TP53* alterations and complex karyotype, with 90% showing monosomal karyotype. As listed in Table 3, treatment included hypomethylating agent (HMA) alone (*n* = 5), HMA + venetoclax (*n* = 12), intensive chemotherapy (*n* = 4), supportive care (*n* = 7), and quizartinib (*n* = 1); no responses or allogeneic stem cell transplants were documented, and all patients died at a median 1.8 months (range 0.2–9.3). Figure 2 illustrates survival data on these 29 patients, stratified by treatment type. PEL was universally fatal with no evidence of survival impact from typical AML therapies, including combination of hypomethylating agents and venetoclax; the 3- and 12-months survival rates were 24% and 0%, respectively. Univariate analysis of multiple host or disease factors failed to identify a statistically significant predictor of survival.

**Table 3:** Clinical and laboratory findings of 29 patients diagnosed with pure erythroid leukemia (PEL), obtained at the time of diagnosis with treatment and survival data.

Variables	All patients
	*N* = 29
Age at diagnosis in years; media*n* (range)	67 (27–86)
Male, *n* (%)	22 (76%)
Hemoglobin g/dL; media*n* (range)	8 (5.9–11.3)
Hemoglobin <10 g/dl, *n* (%)	27 (93%)
Ring sideroblasts, *n* (%)	17 (5%-60%)
Platelets × 10^9^/L; media*n* (range)	18 (4–146)
Platelets <100 × 10^9^/L, *n* (%)	27 (93%)
Dysplastic megakaryocytes, *n* (%)	26 (89%)
Leukocytes × 10^9^/L; media*n* (range)	3 (0.6–31)
ANC x 10^9^/L; media*n* (range)	1.2 (0–5.9)
ANC < 1.5 × 10^9^/L; *n* (%)	18 (62%)
Erythroblasts %; media*n* (range)	90 (50–100)
Blood blast %; media*n* (range)	3 (0–88)
*Cytogenetics:*	
Monosomal karyotype, n (%)	26 (89.6%)
* Other chromosomal abnormalities:*	
Abnormalities of 5, *n* (%)	22 (81%)
Abnormalities of 7, *n* (%)	19 (70%)
Abnormalities of > 10, *n* (%)	22 (81%)
*Somatic mutations:*	
Multi-hit *TP53* mutation, *n* (%)	29 (100%)
Other somatic mutations, *n* (%)	10 (34%)
*JAK2*	3 (30%)
*TET2*	2 (20%)
*NRAS*	1 (10%)
*DNMT3A*	1 (10%)
*IDH2*	1 (10%)
*CSF3R*	1 (10%)
*ASXL1*	1 (10%)
*Immunophenotypic characteristics by flow cytometry positive for, n (%):*	
*CD7*	15 (75%)
*CD117*	21 (91%)
*CD13*	14 (63%)
*CD33*	10 (47%)
*CD36*	21 (95%)
*CD34*	10 (43%)
*CD45*	11 (47%)
*HLA-DR*	4 (20%)
*Immunophenotypic characteristics by immunohistochemistry positive for, n* (%):	
*CD34*	8 (36%)
*CD71*	18 (100%)
*CD117*	20 (90%)
*CD61*	3 (11%)
*E-Cadherin*	21 (91%)
*Glycophorin A*	8 (50%)
*Hemoglobin*	13 (59%)
*Pure erythroid leukemia clinical presentation:*	
Primary, *n* (%)	10 (34%)
Secondary, *n* (%)	10 (34%)
Therapy-related, *n* (%)	9 (31%)
First-line PEL therapy:	
Supportive care, *n* (%)	7 (24%)
Hypomethylating agents alone, *n* (%)	5 (17%)
Hypomethylating agents + Venetoclax, *n* (%)	12 (41%)
Induction chemotherapy, *n* (%)	4 (13%)
Quizartinib, *n* (%)	1 (3%)
*Pure erythroid leukemia therapy response:*	
No remission, *n* (%)	29 (100%)
Allogenic steam cell transplant, *n* (%)	0 (0%)
Overall survival after PEL in months; media*n* (range)	1.8 (0.2–9.3)
Deaths; *n* (%)	29 (100%)
Cause of death: *n* (%)	
Disease progression	26 (90%)
Septic shock and cardiopulmonary arrest	1 (3.3%)
Severe sepsis and shock	1 (3.3%)
Sepsis and disease progression	1 (3.3%)

## Discussion

Herein, we provide a detailed and comprehensive analysis of the pathologic characteristics of 41 cases of pure erythroid leukemia. To our knowledge, this represents the largest series of pure erythroid leukemia cases reported to date. Based on our composite findings, we would argue that pure erythroid leukemia is a unique pathologic entity and should be considered to remain a pathologic diagnosis in hematologic classification systems. While the overall natural disease course may be similar to other acute myeloid leukemias with *TP53* mutation(s), it seems that, in this particular disease, the features are overall highly reproducible and provide a clear indication to the healthcare team as to disease expectations. Given that a diagnosis of pure erythroid leukemia may be challenging from a pathologic perspective, and that the prognostic implication from such a diagnosis is grave (median overall survival 2 months), a comprehensive and systematic approach with the utilization of a diverse set of ancillary tools may be necessary in the elucidation of these rare cases.

Pure erythroid leukemia, in adults, can be summarized with the following features (Fig. 1): presentation with anemia and thrombocytopenia, suboptimal bone marrow aspirate smears/touch preparations, distinctive cytology with predominantly pronormoblasts and minimal presence of maturing erythroid elements, may mimic metastatic disease in the core biopsy (25%), association with ring sideroblasts (70%), cytogenetic complexity (100%) with hypodiploidy and often doubling of that clone, deletion abnormalities of chromosomes 5 and 7 (78% and 66%, respectively), extraordinarily simple NGS mutational profile (71% show no myeloid disease associated gene abnormality aside from *TP53* mutation) and double *TP53* genetic alterations (100%). The immunophenotypic assessment of these cases typically requires a combination of both flow cytometry and immunohistochemistry to confidently determine that the blasts are truly of erythroid lineage.

The differential diagnosis of PEL includes florid reactive erythroid hyperplasias and other myeloid neoplasms with a predominance of erythroid precursors. For the reactive etiologies, we would not expect to identify dysplastic megakaryocytes, a cytogenetic abnormality or an aberrant phenotype (eg. CD7 positivity) on the pronormoblasts. Similarly, strong uniform nuclear expression of TP53 by immunohistochemistry would argue against a non-neoplastic possibility [8]. As for the myeloid neoplasms with an abundance of erythroid precursors, this can be trickier to sort out but if the criteria for PEL are systematically applied, a correct diagnosis is rendered. For example, in acute myeloid leukemia with myelodysplasia-related changes, there are ≥20% myeloid-lineage blasts. In myelodysplastic syndrome, particularly when high grade, one may see a complex abnormal karyotype or monosomal karyotype as well as erythroid predominance, but erythroid precursors should not comprise >80% of bone marrow nucleated elements.

A diagnosis of PEL may be established by assessing for the characteristic distinctive pronormoblastic cytology, variable expression of the erythroid-specific antigens hemoglobin and glycophorin A, and presence of biallelic *TP53* alterations that are always associated with complex/monosomal karyotype [1, 2, 7, 12, 13]. Two new classification systems for hematopoietic tumors have recently been published; in the International Consensus Classification (ICC), PEL is no longer recognized as a separate entity and is instead included in a broader category of AML with mutated *TP53* [3]; in the proposed 5th edition of the WHO document, The WHO alters the terminology from PEL to acute erythroid leukemia (AEL) [4]. The ICC system emphasizes in its category of acute myeloid leukemia that one intention was to “move to a more genetically-defined classification”. The newly defined AML with mutated *TP53*, includes cases with single (or multiple) somatic *TP53* mutation with a variant allele frequency of ≥10% and ≥20% peripheral blood or bone marrow myeloid blasts/blast equivalents, or cases which meet criteria for pure erythroid leukemia. We agree with the findings reported in the literature that AML with mutated *TP53* is typically associated with complex including monosomal karyotype and an overall dismal clinical outcome. That said, it should be remembered that there is underlying premise in the practice of medicine that there is an “art” to what we do as physicians and as members of the health care team as we serve our communities and our patients. We would suggest that in this particular entity, pure erythroid leukemia, there is an art to this diagnosis through a careful assessment of its unique morphology and laboratory, immunophenotypic, genetic and clinical findings. Perhaps this could be included as a qualifier to the diagnosis of AML with mutated *TP53*.

In PEL, the unique cytogenetic and molecular genetic characteristics might suggest therapeutic targets beyond *TP53*; and its invariable association with biallelic *TP53* alterations, implies an even worse diagnosis, compared to single-hit *TP53* abnormalities. The current study also underscores the limited value of AML-like treatment in PEL, including that of HMA + venetoclax combination. We have previously reported the value of HMA + venetoclax in both newly diagnosed and relapsed/refractory AML [14–16]. In one of these reports involving newly diagnosed AML, [14] unlike the case in the current study, responses to HMA + venetoclax were documented in 8 (32%) of 25 *TP53*-mutated AML cases, most of whom expressed monoallelic *TP53* alteration. It is possible that the difference in response rates between the two studies might be related to their difference in *TP53* allelic state. Regardless, the fact that not even one patient responded to any of the AML-like treatment instituted highlights the urgent need for new investigational agents. In a recent multi-center report of 291 patients with *TP53* mutated AML, treated with various induction regimens, complete remission with or without count recovery (CR/CRi), was achieved in only 29%, and 46 patients were bridged to ASCT [17]; in the particular study, HMA + venetoclax combination therapy was the most successful in inducing CR/CRi and outcome was favorably influenced by ASCT and not *TP53* variant allele frequency. These observations suggest the potential value of ASCT in PEL but the current lack of chemotherapy regimens capable of inducing CR/CRi remains to be a challenge.

## Supplementary information

Reproducibility checklist

## Data Availability

The data that support the findings of this study are available from the corresponding author upon reasonable request.
